# Are MTV and TLG Accurate for Quantifying the Intensity of Brown Adipose Tissue Activation?

**DOI:** 10.3390/biomedicines12010151

**Published:** 2024-01-11

**Authors:** Wael Jalloul, Mihaela Moscalu, Roxana Moscalu, Despina Jalloul, Irena Cristina Grierosu, Mihaela Gutu, Danisia Haba, Veronica Mocanu, Mihai Marius Gutu, Cipriana Stefanescu

**Affiliations:** 1 Department of Biophysics and Medical Physics-Nuclear Medicine, “Grigore T. Popa” University of Medicine and Pharmacy, 700115 Iasi, Romania; jalloul.wael@umfiasi.ro (W.J.); despina.prisacariu.dp@gmail.com (D.J.); irena.raileanu@umfiasi.ro (I.C.G.); cipriana.stefanescu@umfiasi.ro (C.S.); 2 Department of Preventive Medicine and Interdisciplinarity, “Grigore T. Popa” University of Medicine and Pharmacy, 700115 Iasi, Romania; 3 Manchester Academic Health Science Centre, Cell Matrix Biology and Regenerative Medicine, The University of Manchester, Manchester M13 9PT, UK; roxana.moscalu@mft.nhs.uk; 4 County Hospital of Emergency “Saint John the New”, 720224 Suceava, Romania; miha.gutu@yahoo.co.uk (M.G.); misugutu@yahoo.fr (M.M.G.); 5 Department 1 Surgery, “Grigore T. Popa” University of Medicine and Pharmacy, 700115 Iasi, Romania; danisia.haba@umfiasi.ro; 6 Department of Morpho-Functional Sciences (Pathophysiology), “Grigore T. Popa” University of Medicine and Pharmacy, 700115 Iasi, Romania; veronica.mocanu@umfiasi.ro

**Keywords:** brown adipose tissue, ^18^F-FDG PET/CT, SUVmax, MTV, TLG

## Abstract

Recent research has suggested that one novel mechanism of action for anti-obesity medications is to stimulate the activation of brown adipose tissue (BAT). ^18^FDG PET/CT remains the gold standard for defining and quantifying BAT. SUVmax is the most often used quantification tool in clinical practice. However, this parameter does not reflect the entire BAT volume. As a potential method for precisely evaluating BAT, we have utilised metabolic tumour volume (MTV) and total lesion glycolysis (TLG) to answer the question: Are MTV and TLG accurate in quantifying the intensity of BAT activation? After analysing the total number of oncological ^18^F-FDG PET/CT scans between 2021–2023, we selected patients with active BAT. Based on the BAT SUVmax, the patients were divided into BAT-moderate activation (MA) vs. BAT-high activation (HA). Furthermore, we statistically analysed the accuracy of TLG and MTV in assessing BAT activation intensity. The results showed that both parameters increased their predictive value regarding BAT activation, and presented a significantly high sensitivity and specificity for the correct classification of BAT activation intensity. To conclude, these parameters could be important indicators with increased accuracy for classifying BAT expression, and could bring additional information about the volume of BAT to complement the limitations of the SUVmax.

## 1. Introduction

Currently, obesity is acknowledged as a real medical condition that demands medication and, in certain situations, surgical intervention [1]. Throughout the past few years, several drugs have been approved for the treatment of obesity; however, both their effectiveness and serious side effects have been documented [2]. Recent research has suggested that one novel mechanism of action for anti-obesity medications is to stimulate the activation of BAT and the transformation of white adipose tissue (WAT) into beige adipose tissue (Beige AT) [2,3]. These effects on fat tissues could be the consequences of various stimuli such as b3-adrenoceptor agonists, physical activity, and low-temperature exposure [1,4,5].

BAT cells contain numerous small lipid vacuoles and well-developed mitochondria. In contrast, WAT cells incorporate a single large lipid vacuole and a few mitochondria [6,7].

In addition, brown fat distribution is predominant in small mammals and infants [8], however, it declines with age and disappears in adulthood [9].

Within brown fat uncoupled mitochondria, fatty acids are “burned” to initiate thermogenesis [10,11].

Apart from its function in maintaining thermal equilibrium (in both severe stress and cold adaptation), BAT is involved in maintaining energy homeostasis [1]. According to Feldmann et al. [12], mice kept at thermoneutral temperatures develop an obese phenotype due to the ablation of the uncoupling protein-1 (UCP-1), which represents the necessary protein for heat production in BAT [11].

Since it produces a range of adipokines, BAT also functions as an endocrine tissue [13,14]. In addition to controlling vascular function and coagulation, BAT-adipokines play a role in the regulation of nutrition behaviour, insulin sensitivity, energy homeostasis, glucose and lipid metabolism, and adipogenesis [15,16,17].

All these features demonstrate BAT’s anti-obesity function, along with its potential to treat metabolic disorders associated with obesity, such as diabetes and cardiovascular disease [3,16].

Beige AT represents a novel subtype of BAT resulting from the browning of WAT [1,18]. Furthermore, the visceral WAT, which is inflammatory in obese patients and may be implicated in obesity-related metabolic diseases, is the target of this type of adipose conversion [1]. In order to develop anti-obesity drugs that activate BAT/transform WAT in beige AT, suitable animal models and imaging techniques need to be used for pharmacological research [1,18].

For a variety of cancer types, positron emission tomography coupled with computed tomography (PET/CT) using the glucose analogue radiotracer 18-fluorodeoxyglucose (^18^FDG) has been utilised for staging, restaging, and treatment response monitoring [9].

This imaging modality continues to be the gold standard technique for defining and quantifying brown fat’s volume of activity, along with BAT’s overall energy expenditure [9,19]. The combination of two essential tissue characteristics on an ^18^FDG PET/CT scan is used to define the presence of active BAT: (1) a fatty tissue with abnormally high ^18^FDG uptake; and (2) a tissue radio-density on CT indicating the presence of adipose tissue [9].

Standardised uptake value (SUV), a ratio of tissue radioactivity concentration reported to the injected dose normalised by body mass (or lean body mass (LBM)), is the most widely used PET parameter for quantifying metabolic activity [20]. The highest SUV pixel value within a region of interest (ROI) is known as the maximum SUV (SUVmax) [20]. Due to its ease of measurement, SUVmax is the most often used quantification tool in clinical practice. However, since this parameter is based on a single pixel, it does not accurately reflect the entire volume’s metabolic burden. Moreover, different patient aspects and imaging parameters affect the SUVmax, which makes it susceptible to image noise [20].

Since then, there have been several approaches developed for using ^18^F-FDG PET to segment tumours. Noticing that the measurement of the tumour volume with a high metabolism is called metabolic tumour volume (MTV), while the product of the average SUV within an ROI (SUVmean) and MTV is known as total lesion glycolysis (TLG) [20,21]. Furthermore, compared to SUVmax, MTV and TLG are recognised as more accurate tools that precisely quantify metabolic tumour burden and, subsequently, have shown essential benefits in various kinds of cancer [22,23,24,25].

The majority of previous studies that investigated BAT used SUVmax as the main quantification tool. In our research, as a potential method for precisely evaluating brown fat, we utilised the volumetric parameters MTV and TLG to answer the question: Are MTV and TLG accurate to quantify the intensity of BAT activation?

This research could be a step towards developing a potential therapeutic method or algorithm for obesity and related metabolic disorders, such as diabetes and cardiovascular disease, based on the activation of BAT/browning WAT.

## 2. Materials and Methods

### 2.1. Patients

The study group included patients referred to the nuclear medicine laboratory of the county hospital of Emergency “Saint John the New” Suceava, Romania, between March 2021 and August 2023 for a variety of oncological diagnoses, such as ovarian cancer, lung cancer, unknown primary cancers, Hodgkin’s lymphoma, non-Hodgkin’s lymphoma, breast cancer, uterine cancer, testicular cancer, ear, nose, and throat (ENT) cancer, pancreatic cancer, gastric cancer, colon cancer, rectal cancer, melanoma, hepatic cancer, and Kaposi’s sarcoma.

**Figure 1:**
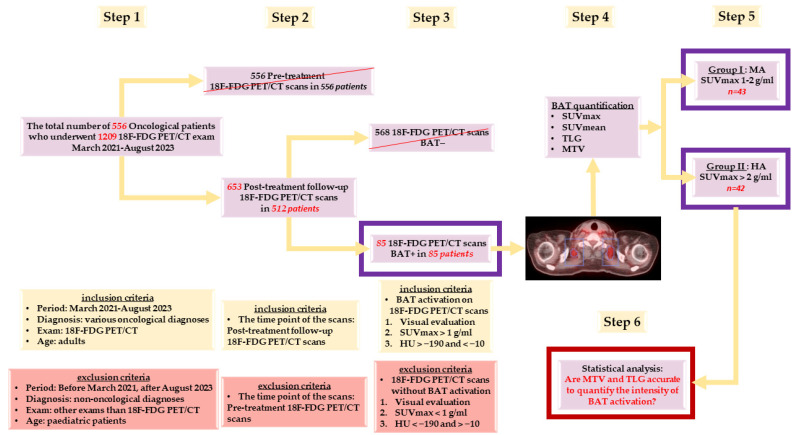
Schematic representation of the research design. MA = moderate activation, HA = high activation.

Based on the exclusion and inclusion criteria, defined in Figure 1, from the total number of 556 examined patients, 85 showed a metabolic activation of brown fat in their ^18^F-FDG PET/CT scans. These cases with active BAT represented the chosen group for analysing the activation intensity of brown fat (Figure 1).

The chosen BAT+ scans were performed for post-treatment follow-up, and the selected patients showed partial or complete responses to the treatment. In order to prevent potential treatment effects on ^18^F-FDG biodistribution, including inflammation or infection, and according to national and European guidelines, the chosen scans were carried out at least 2–4 weeks after the last cycle of chemotherapy, 2–3 months after radiotherapy, or 6 weeks after surgery [26].

Taking into consideration their potential influence on ^18^F-FDG BAT activity, we descriptively assessed the patients’ demographic and anthropometric factors, including, age, gender, body mass index (BMI), cancer diagnosis, and cancer-related therapies (chemotherapy, radiotherapy, surgery).

Every stage of the procedure was completed in accordance with the institutional recommendations. Prior to each examination, patients had provided informed consent for the possible use of their medical data for research. The approval of the hospital’s medical ethics committee was obtained for the use of these data from the laboratory archive. As a retrospective and anonymous study, no additional ethical consent was required for this investigation.

### 2.2. ^18^F-FDG PET/CT Scanning Protocol

The entire procedure was carried out in compliance with the European Association of Nuclear Medicine’s (EANM) practice guidelines for ^18^F-FDG PET/CT tumour imaging [26].

The patients fasted for at least six hours before the scan. To ensure that BAT expression was unaffected by external temperature fluctuations, the patients were kept in our laboratory in thermoneutral conditions (22–24 °C) throughout the whole process.

Blood glucose levels, which ranged between 65 and 152 mg/dL (including patients with Diabetes), were measured prior to the IV administration of ^18^F-FDG (dose interval: 153–523 MBq). Throughout the injection and subsequent uptake phase, the patients were kept silent, seated or supine, to minimise the amount of radiotracer uptake in their muscles. About an hour after the administration of ^18^F-FDG, the imaging acquisition began.

The patients were usually positioned with their arms raised and supported above their heads to avoid both artefacts caused by reducing the measured field of view (FOV) and beam-hardening artefacts in the abdomen and pelvis. For most oncological pathologies, the scan’s exposure of the region between the vertex of the skull and mid-thigh was suitable. Patients who had tumours with suspected distant metastases that were likely to spread to other parts of the body, including the head, skull, brain, and lower extremities (such as melanoma, sarcomas, and myeloma), underwent complete whole-body scans according to European recommendations [26].

All of the scans were conducted using the GE Discovery IQ system. The scanning protocol involved a topogram, a low-dose CT scan for attenuation correction (AC) and anatomical correlation, and a whole-body standard CT reconstruction followed by PET acquisition with and without AC. The acquisition parameters such as tube current-voltage (max 140 kV), slice thickness (2.5 cm), rotation time, and pitch were selected based on the reason for conducting the CT scans. The PET data were obtained with an acquisition time of 2 min/bed and 8-bed positions.

### 2.3. Image Processing and Interpretation

To accurately study and identify the active BAT pattern, a set of steps and criteria were implemented to ensure that bias was reduced and precision was improved in the analysis.

#### 2.3.1. Visual Determination of BAT Activation

Taking into account the characteristic distribution of brown fat, as well as the areas of physiological/pathological ^18^F-FDG uptake [27,28], two nuclear medicine physicians analysed the total number of 1209 oncological ^18^F-FDG PET/CT scans (certain patients underwent multiple ^18^F-FDG PET/CT exams to evaluate the effectiveness of their treatments).

The aim was to select the images that presented BAT activation. To resolve a possible disagreement, a third nuclear medicine physician was consulted.

#### 2.3.2. Quantitative Confirmation of BAT Activation

After the visual determination of active BAT presence in the scans, the physicians verified two important aspects:

- Areas with active BAT had a radio-density corresponding to fat tissue in CT imaging (Hounsfield units between −10 and −190) [27];
- SUVmax (LBM) in BAT was higher than 1 g/mL [29,30].

#### 2.3.3. Quantification of BAT Activation Intensity in Every BAT+ Scan

We drew a volume of interest (VOI) in every brown fat localisation. Since there is no consensus on the most appropriate way to define the VOI [31], we used the fixed relative threshold (defined as a certain percentage of the SUVmax) of 42%, which represents one of the most widely accepted thresholds for its predictive value [32,33,34]. This fixed relative threshold method was utilised for a precise automatic delineation of BAT volume and accurate measurement of quantitative PET parameters [20,25,32,35]. Moreover, the VOI was drawn at a sufficient distance from visible lymph nodes, which represent potential findings in lymphoma patients [27,36].

**Figure 2:**
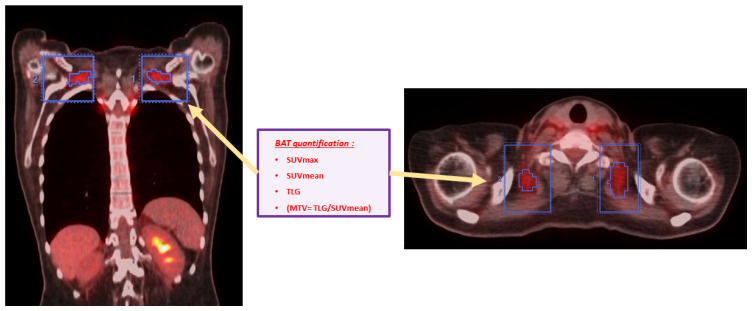
Delineation of BAT volume and measurement of the quantitative PET parameters in ^18^F-FDG PET/CT scans in our patients.

The SUVmax (LBM), SUVmean, and TLG in each BAT+ VOI were measured automatically using the software AW VolumeShare7—General Electric. Knowing that TLG = MTV × SUVmean, the MTV was calculated by the ratio between the TLG and SUVmean (MTV = TLG/SUVmean) [20,25,32,35] (Figure 2).

The measurements of total BAT activity and volume in every BAT + scan were carried out as follows:

- **Total BAT SUVmax in g/mL (Tot SUVmax)** = We chose the greatest value of SUVmax measured in all of the VOIs drawn from all BAT localisations;
- **Total BAT SUVmean in g/mL (Tot SUVmean)** = We calculated the mean of all SUVmean values measured in all of the VOIs in all BAT localisations;
- **Total BAT TLG in cm^3^ × g/mL (Tot TLG)** = We calculated the sum of all TLG values measured in all of the VOIs drawn from all BAT localisations;
- **Total BAT MTV in cm^3^ (Tot MTV)** = We calculated the sum of all MTV values measured through the ratio of Tot TLG/Tot SUVmean in all of the VOIs drawn from all BAT localisations.

The obtained values were used in the statistical analysis.

**Figure 3:**
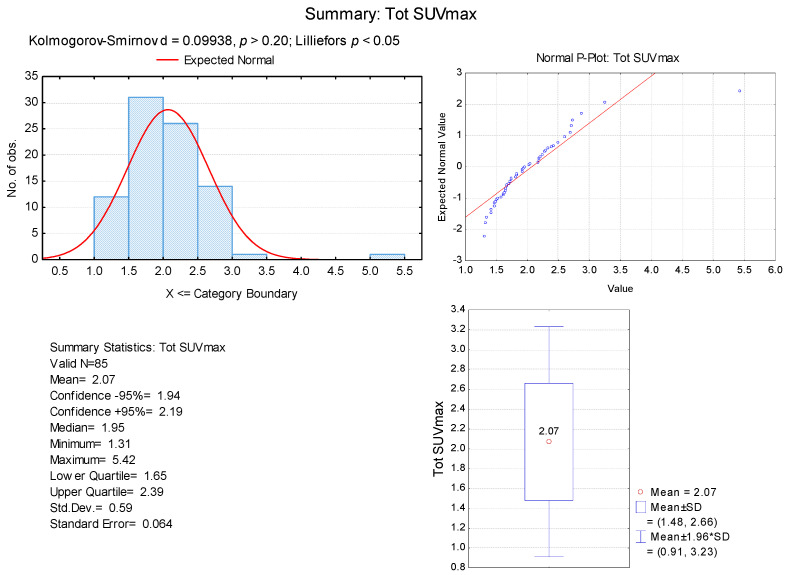
Histogram of Tot SUVmax values.

Since SUVmax has always been used in previous studies as the reference parameter for measuring BAT activity, we created a histogram to display the distribution of the Tot SUVmax values obtained (Figure 3).

The Tot SUVmax measurements showed a mean value of 2.07 ± 0.95 g/mL and a median value of 1.95 g/mL. We also noticed that the Tot SUVmax values represented a normal distribution. Taking into account these findings, we considered the Tot SUVmax of 2 g/mL as a reference to appreciate the intensity of BAT activation.

Furthermore, based on these features (Figure 3), we classified our selected patients into two groups according to the intensity of BAT activation (Figure 1):

- **Group I:** Patients with BAT moderate activation (MA)—Tot SUVmax between 1–2 g/mL;
- **Group II:** Patients with BAT high activation (HA)—Tot SUVmax > 2 g/mL.

Furthermore, we statistically analysed the accuracy of Tot TLG and Tot MTV in quantifying the intensity of brown fat activation.

### 2.4. Statistical Analysis

STATA 16 software (StataCorp LLC, 4905 Lakeway Drive, College Station, TX, USA) and SPSS v.29 (IBM Ireland Product Distribution Limited, IBM House, Shelbourne Road, Ballsbridge, Dublin, Ireland) were used for all of the statistical analyses.

Continuous variable types were reported as means ± standard deviation (SD) or medians (range). The Kolmogorov–Smirnov test was applied to verify the normal distribution of the variables. The qualitative variables were presented as absolute frequency (*n*) and relative frequency (%). The accuracy of the predictive power of Tot TLG and Tot MTV in assessing the intensity of BAT activation (patients with BAT MA/patients with BAT HA) was evaluated based on the receiver operating characteristic (ROC) curve, taking into account the area under the curve (AUC), representing the sensitivity (Se) and specificity (Sp). The Tot SUVmax value with a cutoff of 2 g/mL was considered as a reference.

Validation of the prediction model based on Tot MTV and Tot TLG of BAT activation was also carried out using precision-recall curves, which evaluate the prediction accuracy based on the considered parameters.

Regression analysis was used to look for potential correlations between Tot SUVmax and Tot MTV, as well as between Tot SUVmax and Tot TLG.

The significance level calculated in the utilised tests (*p*-value) was considered significant for values of *p* < 0.05.

## 3. Results

Based on the Tot SUVmax values, among the selected 85 patients (15.29% of all the included patients) who presented active brown fat in 85 ^18^F-FDG PET/CT scans (7.03% of all the included images), 43 patients (50.6%) showed BAT MA, and 42 patients (49.4%) had BAT HA (Table 1).

The mean age of the patients was 64.2 ± 12.4 years (group I: MA; 66.7 ± 5.1 years, group II: HA; 61.7 ± 15.3 years). The proportion of active BAT in males was higher than in females (70.6%), and there was no gender-specific differences in the Tot SUVmax groups (*p* = 0.432).

The mean BMI was 25.9 ± 4.81 kg/m^2^ (MA: 26.1 ± 5.1 vs. HA: 25.8 ± 4.5), indicating no significant difference (*p* = 0.961) between the groups.

Six individuals were already known diabetics with controlled blood sugar levels, presenting normal glucose values prior to ^18^F-FDG PET/CT examination.

None of the patients we selected were receiving treatment with beta-adrenergic receptor agonists or blockers.

Considering the oncological diagnostic type, no significant difference was found between the Tot SUVmax groups (*p* = 0.5).

Patients received surgical resections in 54.1% of the cases; out of these, 18.8% underwent adjuvant chemotherapy, and 21.2% received both chemotherapy and radiotherapy. No noteworthy distinction regarding the treatment strategy was observed between the groups (*p* = 0.45).

**Figure 4:**
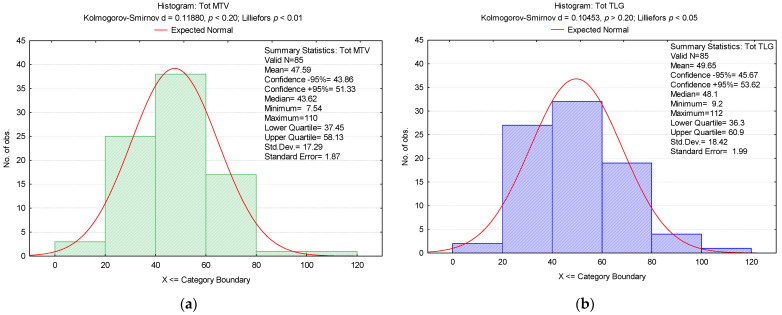
Histogram of (**a**) Tot MTV and (**b**) Tot TLG values.

The mean value (mv) of Tot MTV was 47.59, and for Tot TLG it was 49.65. The Tot MTV confidence interval (CI) was 43.87–51.33, and for Tot TLG it was 45.67–53.62 (Figure 4).

**Figure 5:**
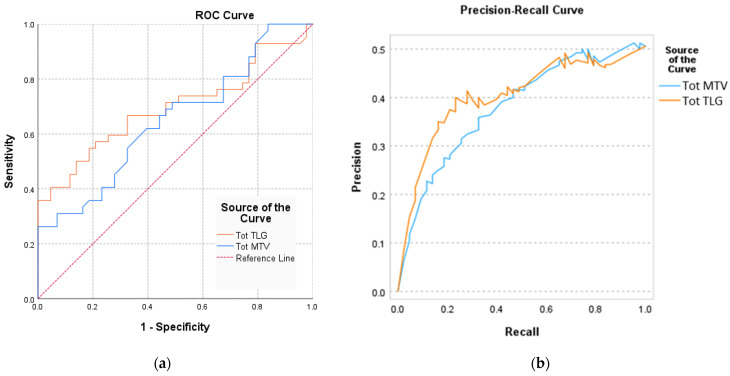
The predictability of Tot MTV and Tot TLG considering BAT activity, based on (**a**) ROC curves and (**b**) precision-recall curves.

Using ROC curves, the study analysed the accuracy predictive power of Tot MTV and Tot TLG on patient classification based on BAT activation (patients with BAT MA/patients with BAT HA) (Figure 5a). The global accuracy of the prediction method was described by the AUC (Table 2 and Figure 5a).

The results show that both the Tot MTV ((AUC (95%CI)_MTV_ = 0.694 (0.579–0.809), *p* = 0.001)) and Tot TLG ((AUC (95%CI)_TLG_ = 0.721 (0.654–0.832), *p* = 0.013)) increased the predictive value for the estimation of BAT activation (Table 2 and Figure 5a).

Based on the Tot MTV and Tot TLG values, precision-recall estimates the number of correctly classified results, assesses the prediction success, and measures their relevance.

Our curves showed a large area under the curve that represents both high recall and high precision; high precision indicates a low false positive rate, and high recall refers to a low false negative rate (Figure 5b).

The Tot MTV and Tot TLG values showed significantly increased sensitivity (Se_MTV_ = 79%, 95%CI: 67–91%; Se_TLG_ = 89%; 95%CI: 71–92%) (*p* < 0.01) for the correct classification of patients with activated BAT. Also, the specificity of Tot MTV (Sp_MTV_ = 75%, 95%CI: 69–87%) and Tot TLG (Sp_TLG_ = 76%, 95%CI: 68–94%) parameters had increased values, which indicate that these parameters could be important indicators with increased accuracy for classifying the intensity of BAT activation into MA or HA (Table 3).

**Figure 6:**
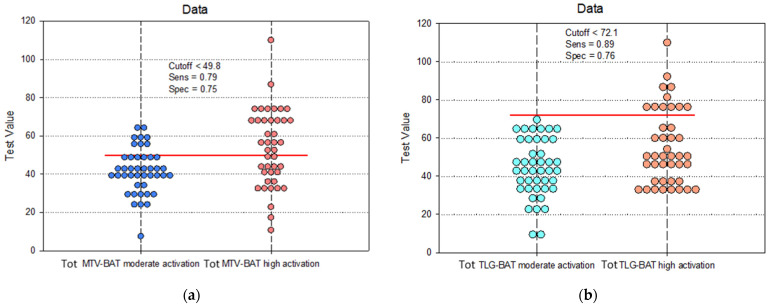
Paired histogram for estimating the cutoff values of the (**a**) Tot MTV and (**b**) Tot TLG, considering the predictability of BAT activity.

For optimal values of Se and Sp, the cutoffs for Tot MTV and Tot TLG were established (Figure 6, Table 3) so that cases could be classified into BAT HA or MA. Thus, for a Tot MTV greater than 49.8, there is a significantly increased probability that these cases present BAT HA. Also, for a Tot TLG higher than 72.1, the patients can be considered with BAT HA. The statistical parameters calculated for these cutoff values indicate an increased accuracy in classifying the BAT activation level (Figure 6).

These results confirmed that Tot MTV and Tot TLG have increased accuracy in classifying patients into BAT HA or MA.

**Figure 7:**
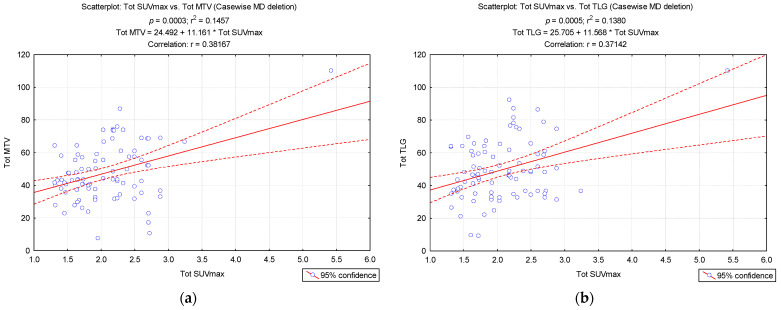
Correlations between (**a**) Tot MTV and Tot SUVmax, as well as between (**b**) Tot TLG and Tot SUVmax.

The regression analysis between Tot SUVmax and Tot MTV (r = 0.381, *p* = 0.0003), as well as between Tot SUVmax and Tot TLG (r = 0.371, *p* = 0.0005) showed a significant positive correlation for the two volumetric parameters with Tot SUVmax values (Figure 7).

## 4. Discussion

Considering that BAT is involved in the clearance of plasma triglycerides and glucose homeostasis [37,38,39], Vosselman et al. stated that these findings demonstrate BAT’s anti-obesity function and its potential to treat metabolic disorders associated with obesity in rodents, such as diabetes and cardiovascular disease [40].

Wang et al. [41] managed to establish an experimental protocol for BAT functional imaging in mice using ^18^F-FDG to standardise the imaging processes and to bring uniformity across post-image analysis. Reliable quantification of the BAT activation intensity using PET volumetric parameters MTV and TLG will complete this method and make it more precise to assess drugs/compounds that modulate BAT activity. Consequently, the resulting technique may contribute to the development of a therapeutic approach or algorithm for obesity and associated metabolic disorders, including diabetes and cardiovascular disease, based on the induction of this fat tissue.

In adult humans, the existence of functional BAT was primarily recognised through the application of ^18^FDG PET/CT [9,42]. When it comes to glucose metabolism, it appears that the very high metabolic activity of normally metabolically inactive fat tissue is what finally convinced the scientific community to recognise BAT as an organ of interest for energy balance, along with its possible therapeutic effects on obesity and type 2 diabetes [9,14,43].

Since ^18^F-FDG’s distribution accurately represents the metabolic rate in the body, it is considered the most pertinent radiotracer for research on BAT metabolism [1,19].

Even though BAT thermogenic activity is not well reflected by BAT glucose metabolism, ^18^FDG PET/CT is still regarded as the “gold standard” technique for defining the presence and measuring brown fat volume in humans [27,44,45,46,47,48,49,50].

Due to its high reproducibility and simplicity of measurement as a semiquantitative parameter, SUVmax is the most frequently used ^18^F-FDG PET/CT parameter [32]. This tool is highly correlated with histopathological grade and clinical outcomes [25]. Nevertheless, SUVmax is susceptible to significant factors, as it is only a single-pixel value within a ROI [32]. Furthermore, SUVmax may not accurately reflect the overall characteristics of the entire ROI; rather, it only illustrates the section of the ROI that is most active [22]. Consequently, SUVmax is unlikely to be a reliable indicator of the metabolic activity of heterogeneous tumours and multilocation-activated BAT [32]. Considering its non-homogeneity, along with the diversity of shape and localisation of BAT in some cases, SUVmax cannot precisely quantify the brown fat metabolic expression.

Since MTV and TLG represent both the total tumour burden and the tumour’s glucose metabolism level, they overcome these SUVmax limitations, and are therefore considered to be more accurate prognostic indicators [51].

Our findings showed that both MTV and TLG have increased predictive value for the estimation of BAT activation. In accordance with our results, it has already been demonstrated that these tools have a prognostic significance in several cancers [20]. Various studies determined that these PET parameters have an impact on prognoses in head and neck cancer [52], as well as in non-small cell lung cancer [20,53], osteosarcoma [54], and pancreatic ductal adenocarcinoma (PDAC) [32].

In our study, MTV and TLG presented a significantly increased sensitivity and specificity for the correct classification of BAT activation intensity. Consequently, these parameters could be important indicators with increased accuracy for classifying BAT expression into MA or HA. Additionally, these tools had a similar specificity (Sp_TLG_ = 76% vs. Sp_MTV_ = 75%); however, TLG had better sensitivity (Se_TLG_ = 89% vs. Se_MTV_ = 79%). This finding is consistent with previous studies in non-small-cell lung cancer, ovarian cancer, head-and-neck cancer, and osteosarcomas, which have also found that TLG is a more accurate predictor than MTV [22,35,55,56,57,58].

Numerous research studies that compared the reproducibility of these volumetric parameters demonstrated that in head-and-neck cancer, non-small-cell lung cancer, and epithelial ovarian cancer, MTV and TLG have superior prognostic value compared with SUVmax [15,16,17,55,56,57]. Additionally, Choi et al. showed that in soft-tissue sarcoma (STS), TLG is a better predictor of disease-free survival than SUVmax [25].

In contrast, SUVmax was shown to be the most accurate predictive parameter according to a study that examined the tumour response and metabolic parameters in STS patients [59]. Furthermore, Tatewaki et al. deduced that SUVmax was a crucial element in determining the prognosis of PDAC [32]. Additionally, volumetric data such as MTV may enhance the precision of PDAC prediction [32]. In our research, the significant positive correlation of the two volumetric parameters with SUVmax values, along with the important accuracy of these tools, showed that MTV and TLG could bring additional information about the volume of BAT, and can be used as predictive parameters for the intensity of BAT. These findings complement the limitations of SUVmax; however, further studies should be performed to compare the accuracy power of MTV and TLG with SUVmax.

Regarding the confirmation of BAT activation, various SUVmax thresholds were used in previous studies, such as SUVmax > 1 [29,30] and > 1.2 [27]. In our research, considering the distribution of BAT in our patients, SUVmax > 1 was better to confirm the visual determination of BAT expression. Furthermore, no established SUVmax cutoff was described in the literature to classify the intensity of BAT activation. In our study, the distribution of SUVmax values indicated that the most suitable cutoff to categorise the BAT activation into MA or HA was 2. Based on this SUVmax cutoff, we find that an MTV greater than 49.8 and a TLG higher than 72.1 represent potential values to indicate BAT HA.

MTV and TLG have not yet been integrated into routine clinical practice. This is mostly due to the lack of accordance on the best technique for segmenting tumours in FDG PET images [20,60,61]. In contrast to SUVmax, these measurement tools necessitate an exact delineation of the tumour [20].

In order to identify the limits of the VOI, threshold-based techniques have been suggested and assessed. Two approaches are frequently used in clinical practice to determine MTV and TLG, such as the fixed-relative threshold of the SUVmax, along with the fixed-absolute threshold method [62].

Relative thresholds are expressed as a specific percentage of a tumour’s SUVmax. The most commonly recognised predictive values are 40% and 42% [32,34]. Considering these data, and for a suitable delineation of active BAT, we used the threshold of 42%.

Absolute SUV thresholds indicate that any voxel in the VOI having an SUV above the fixed absolute threshold value is classified as a tumour, while voxels below the threshold are regarded as background [32]. As a result of its consistently high prognostic prediction, SUV2.5 is the most commonly used absolute threshold [34,63].

Currently, there is still no confirmation of the precise threshold values and methods linked to prognosis. Tumours with various degrees of uptake and dimensions can be more accurately segmented using adaptive or algorithm-based methods, whereas fixed absolute and relative thresholds have demonstrated obvious disadvantages in this type of malignity [20].

The process of lesion detection and delineation is now more feasible due to recent advancements in commercial software. Increased reproducibility and, consequently, improved clinical accuracy and usefulness are the outcomes of these developments. The Food and Drug Administration (FDA) has approved a software to calculate TLG and MTV. In order to enable meaningful data comparisons in clinical research and trials, international standards for image acquisition and lesion delineation should be established [35]. Afterwards, future findings will complete the brown adipose reporting criteria in imaging studies (BARCIST 1.0) that have been established to standardise ^18^FDG-PET/CT BAT imaging in humans [19,27].

It is important to consider a number of limitations when analysing the results of this study. The small number of patients included in the study groups can be explained by the necessity of selecting only individuals with active BAT, in order to quantify the intensity of this tissue’s activation. Since BAT activation in adults appears in very specific circumstances, these patients constitute a minority. To validate the hypotheses, particular statistical tests were employed, taking into account the limitations of small samples. Consequently, in the univariate statistical analysis that was used for comparisons, the estimates’ statistical power was kept at a respectable level.

The uncommon location and frequent asymmetry of hypermetabolic BAT in the upper abdomen and mediastinum increase the likelihood of a false positive for nodal metastases or primary cancer in these regions. Therefore, our two doctors performed a thorough evaluation of BAT based on all provided scans and CT images (with the assistance of a third doctor to resolve any disagreements in certain cases).

The utility of ^18^F-FDG PET/CT has been amply demonstrated in prior human studies; however, this technique still has a number of important drawbacks, including response heterogeneity, sensitivity to experimental or environmental factors, insensitivity to fatty acid-mediated metabolism (BAT’s preferred energy source), and confounding variables for SUV-based and volumetric quantitation [46]. Therefore, more prospective studies with larger samples and specific methodologies, along with the development of standardised techniques for PET quantification, would help to produce more conclusive results.

## 5. Conclusions

The study answered the research question by showing that MTV and TLG have increased predictive value regarding the estimation of BAT activation, and can be considered as suitable tools for assessing BAT expression intensity.

The significant positive correlation of the two volumetric parameters with SUVmax confirmed that MTV and TLG could bring additional information about the volume of BAT to complement the limitations of SUVmax. However, further studies should be performed to compare the accuracy power of MTV and TLG with SUVmax.

## Figures and Tables

**Table 1:** The demographic and clinical characteristics of patients with oncological pathologies and active BAT.

**Clinical Characteristics**	**Total Patients** ***n*** **= 85**	**MA** ***n*** **= 43**	**HA** ***n*** **= 42**	***p*** **-Value**
Age, median (IQR), years	67 (60–73)	67 (61–74)	68 (53–72)	0.391
Age, mean (SD), years	64.2 ± 12.4	66.7 (8.1)	61.7 (15.3)	
Gender, female/male, *n* (%)	25 (29.4)/60 (70.6)	11 (25.6)/32 (74.4)	14 (33.3)/28 (66.7)	0.432
BMI, median (IQR), kg/m^2^	25.8 (22.7–29.8)	25.2 (21.9–30.4)	26 (23.3–27.9)	0.961
BMI, mean (SD), kg/m^2^	25.9 (4.81)	26.1 (5.1)	25.8 (4.5)	
**Diagnostic, n (%)**				
Melanoma	5 (5.9)	3 (7)	2 (4.8)	0.501
Non-Hodgkin’s lymphoma	1 (1.2)	0 (0)	1 (2.4)	
Hodgkin’s lymphoma	3 (3.5)	1 (2.3)	2 (4.8)	
Kaposi’s sarcoma	1 (1.2)	1 (2.3)	0 (0)	
ENT cancer	12 (14.1)	6 (14)	6 (14.3)	
Lung cancer	18 (21.2)	13 (30.2)	5 (11.9)	
Esophageal cancer	2 (2.4)	1 (2.3)	1 (2.4)	
Pancreatic cancer	3 (3.5)	1 (2.3)	2 (4.8)	
Gastric cancer	3 (3.5)	1 (2.3)	2 (4.8)	
Hepatic cancer	2 (2.4)	1 (2.3)	1 (2.4)	
Colon cancer/Rectal cancer	19 (22.4)	9 (20.9)	10 (23.8)	
Breast cancer	3 (3.5)	1 (2.3)	2 (4.8)	
Testicular cancer	2 (2.4)	1 (2.3)	1 (2.4)	
Uterine cancer	5 (5.9)	3 (7)	2 (4.8)	
Ovarian cancer	2 (2.4)	0 (0)	2 (4.8)	
Unknown primary cancers	4 (4.7)	1 (2.3)	3 (7.1)	
**Treatment, n(%)**				
**with surgical treatment**	46 (54.1)	25 (58.1)	21 (50)	0.451
chemotherapy	16 (18.8)	8 (18.6)	8 (19)	
radiotherapy	1 (1.2)	0 (0)	1 (2.4)	
chemotherapy + radiotherapy	11 (12.9)	7 (16.3)	4 (9.5)	
**without chemotherapy/radiotherapy**	18 (21.2)	10 (23.3)	8 (19)	
without surgical treatment	39 (45.9)	18 (41.9)	21 (50)	
chemotherapy	30 (35.3)	15 (34.9)	15 (35.7)	
radiotherapy	1 (1.2)	1 (2.3)	0 (0)	
chemotherapy + radiotherapy	8 (9.4)	2 (4.7)	6 (14.3)	

**Table 2:** The predictive/accuracy values of Tot MTV and Tot TLG.

	Area under the Curve AUC (95%CI)	Std. Error	*p*-Value
Tot MTV	0.694 (0.579–0.809)	0.059	0.001 *
Tot TLG	0.721 (0.654–0.832)	0.062	0.013 *

(*) Marked effects are significant at *p* < 0.05.

**Table 3:** Sensitivity and specificity of Tot MTV and Tot TLG in predicting BAT activity.

	Sensitivity	95% CI	Specificity	95% CI	PPV	NPV	Cutoff
Tot MTV	0.79	0.67 to 0.91	0.75	0.69 to 0.87	0.85	0.83	49.8
Tot TLG	0.89	0.71 to 0.92	0.76	0.68 to 0.94	0.73	0.87	72.1

CI—confidence interval, PPV—positive predictive value, NPV—negative predictive value.

## Data Availability

The data presented in this study are available on request from the corresponding author.

## Abbreviations

Brown adipose tissue **(BAT)**, white adipose tissue **(WAT)**, beige adipose tissue **(Beige AT)**, uncoupling protein-1 **(UCP-1)**, positron emission tomography/computed tomography **(PET/CT)**, 18-fluorodeoxyglucose **(^18^FDG)**, standardised uptake value **(SUV)**, lean body mass **(LBM)**, region of interest **(ROI)**, the maximum SUV **(SUVmax)**, metabolic tumour volume **(MTV)**, average SUV **(SUVmean)**, total lesion glycolysis **(TLG)**, ear, nose, and throat **(ENT)**, moderate activation **(MA)**, high activation **(HA)**, body mass index **(BMI)**, field of view **(FOV)**, attenuation correction **(AC)**, volume of interest **(VOI)**, total SUVmax **(Tot SUVmax)**, total SUVmean **(Tot SUVmean),** total TLG **(Tot TLG)**, total MTV **(Tot MTV)**, standard deviation **(SD)**, receiver operating characteristic **(ROC)**, area under the curve **(AUC)**, sensitivity **(Se)**, specificity **(Sp)**, mean value **(mv)**, confidence interval **(CI)**, positive predictive value **(PPV)**, negative predictive value **(NPV)**, pancreatic ductal adenocarcinoma **(PDAC)**, Food and Drug Administration **(FDA)**.
